# Immune-cognitive system connectivity reduces bumblebee foraging success in complex multisensory floral environments

**DOI:** 10.1038/s41598-018-24372-5

**Published:** 2018-04-13

**Authors:** Melissa W. Mobley, Robert J. Gegear

**Affiliations:** Department of Biology and Biotechnology, Worcester Polytechnic Institute, Worcester, 01609-2280 USA

## Abstract

Bumblebees are declining at alarming rate worldwide, posing a significant threat to the function and diversity of temperate ecosystems. These declines have been attributed, in part, to the direct effect of specific pathogens on bumblebee survival. However, pathogens may also have a negative impact on host populations indirectly through immune-induced cognitive deficits in infected individuals. To gain greater insight into mechanisms and potential conservation implications of such ‘immune-brain crosstalk’ in bumblebees, we non-pathogenetically activated humoral and cellular immune pathways in individuals and then tested for long-term reductions in cognitive performance and foraging proficiency. We show that chronic activation of humoral, but not a cellular, immune pathways and effectors in foragers significantly reduces their ability to flexibly and efficiently harvest resources in multi-sensory floral environments for at least 7 days post-treatment. Humoral defense responses thus have the potential to confer significant foraging costs to bumblebee foragers over timeframes that would negatively impact colony growth and reproductive output under natural conditions. Our findings indicate that fitness effects of immune-brain crosstalk should be considered before attributing wild bumblebee decline to a particular pathogen species.

## Introduction

Bumblebees are declining in abundance, species richness, and geographic distribution at an unprecedented rate worldwide^1–4^. Although the specific cause of these declines is currently unknown, increased exposure to native and/or exotic pathogenic organisms is widely thought to be a significant contributing factor^4–7^. While there is overwhelming evidence that bumblebee pathogens have become more prevalent and diverse over the past decade^6,8–11^, the relationship between specific pathogenic agents and decreases in wild bumblebee populations remain speculative. Given the ecological, economic, and social importance of bumblebees as pollinators^12^, it is therefore imperative that we gain greater mechanistic and functional insight into how infection with different types of pathogen affects bumblebee fitness.

Past studies have shown that infection by some pathogens can impair cognitive processes (i.e. brain functions involved in the acquisition, storage, manipulation of information^13^) needed for bumblebee foragers to adaptively exploit floral resources^14,15^. Because these pathogens do not make contact with the central nervous system, destroy neurons, or have the metabolic capacity to secrete neuromodulators, Gegear *et al*.^15^ proposed that cognitive deficits associated with infection were mediated by some form of communication between the immune system and the brain of host bees rather than by the direct action of the pathogen itself, as has been demonstrated previously in vertebrate systems^16–19^. In support of this ‘immune-brain crosstalk’ hypothesis, Mallon and colleagues subsequently showed that non-pathogenic stimulation of the immune system can reduce performance of restrained and free-flying bees on a simple cognitive task^20–22^. However, mechanisms and potential conservation implications of immune-brain crosstalk in bumblebees have yet to be fully explored.

Bumblebees, like other insect pollinators, defend against pathogens through an innate immune system comprised of humoral and cellular branches^23–25^. Humoral immune responses involve the production of antimicrobial peptides (AMPs) in the fat body and their subsequent release into the hemolymph. Two well-characterized pathogen-dependent signaling pathways regulate the expression of AMP genes in bees^26^. The Toll pathway is primarily activated by fungal and gram positive bacterial infections whereas the immune deficiency (Imd) pathway is activated primarily by gram negative bacterial infections. In contrast, the cellular branch of the insect innate immune system, triggered by wounding or parasitic bodies, is characterized by the activation and proliferation of hemocytes and the production of phenoloxidase, leading to phagocytosis, nodulation, and encapsulation of the pathogenic agent^27^.

At the molecular level, several cellular and humoral immune effectors have been identified in insects that have the potential to alter cognitive behavior in bumblebees. For example, cellular signaling pathways involve the synthesis of biogenic amines such as octopamine and dopamine^27–29^, which are known to play an integral mechanistic role in modulating the insect cognition system^30,31^. There is also evidence that insect humoral (Toll pathway specifically) and cellular responses involve the production of cytokines^32^ and prostaglandins^33^, which are well-known to impair cognitive functioning in humans and other vertebrates^34–36^. Finally, recent genetic studies have shown that AMPs have the capacity to alter neuroactivity, cause neurodegeneration, and reduce memory capacity in *Drosophila*^37–40^.

In this study, we combine behavioral and molecular approaches to determine whether cellular or humoral or both immune responses can impair adaptive decision-making processes in bumblebee foragers over ecologically-relevant timeframes. Specifically, we non-pathogenically elicited cellular (injection with a sterile synthetic ‘Elastomer’ polymer) and humoral (injection with lipopolysaccharide or ‘LPS’) defense responses in foragers, and then measured their performance relative to controls (foragers injected with insect Ringer’s solution) on an ecologically-relevant cognitive paradigm at increasing time points post-treatment. In the paradigm, individual foragers searched for sucrose rewards on a mixed floral array that either 1) forced them to perform a simple color or odor discrimination task (single task experiment) or 2) allowed them to freely choose between performing color, odor, or both discrimination tasks (multitasking experiment). The multitasking experiment was designed to present foragers with the much more cognitively challenging task of managing and rapidly switching between relevant floral information from different sensory modalities (visual and olfactory) in order to maximize their rate of reward intake.

Our behavioral experiments revealed that a single LPS-challenge reduces cognitive functioning and foraging proficiency of bumblebees for at least 7 days. Given that colony growth and reproductive output (fitness) is directly related to the nectar collection rate of foragers in bumblebees and other social bees^41^, these results indicate that immune-cognitive system connectivity has the potential to negatively impact wild bumblebee populations in areas with a high prevalence of certain types of pathogen. However, this scenario assumes that (1) our Elastomer (cellular) challenge did not activate humoral immune pathways and effectors to the same degree as our LPS challenge, and (2) our LPS challenge results in the persistent production of the humoral immune effectors for a period of at least 7 days. To confirm that these assumptions were met, we conducted an additional set of experiments in which qPCR analysis was used to compare whole body gene expression levels of the four known bumblebee AMPs (abaecin, defensin, apidaecin, and hymenoptaecin^42^) between immune-challenged and control bees at 1, 2, 5 and 10 days post-treatment. Although it has been previously demonstrated that LPS challenge, which simulates infection by gram negative bacteria, can increase antimicrobial activity in a different bumblebee species (*B*. *terrestris*) for extended time periods^43^, it is currently unknown whether such chronic activity also occurs in *B. impatiens*. Moreover, it has yet to be determined whether LPS challenge induces chronic expressional of all or a specific subset of AMPs in bumblebees. Filling these knowledge gaps is a critical first step in identifying molecular mechanisms underlying immune-brain crosstalk in bumblebees.

## Results

### Behavioral Experiments

#### Single task experiment

**Figure 1:**
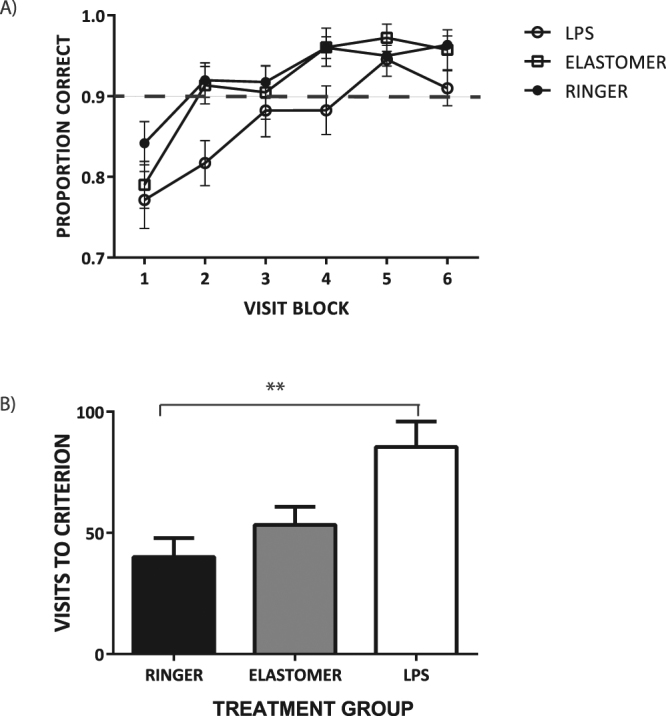
LPS challenge impairs performance of bumblebee foragers on simple stimulus discrimination learning tasks. (**A**) The proportion of rewarding flower types selected by treated bees per block of 20 consecutive flower visits to the single task array. Filled circles = mean for Ringer bees; Open circles = mean for LPS bees; Open squares = mean for Elastomer bees. Dotted line represents task performance threshold of 90% visits to the rewarding flower type. (**B**) Mean number of flower visits required for treated bees to reach 90% task performance threshold over three consecutive blocks of 20 flower visits. Means are shown +/−SE. **P < 0.01.

Elastomer, LPS and Control (hereafter referred to as Ringer) bees all showed an increase in the number of visits to the rewarding flower type as they gained foraging experience on the single task floral array (Fig. 1A; F_5, 95_ = 11.99, p < 0.0001), indicating that immune treatment did not reduce the capacity of foragers to learn simple color and odor discrimination tasks. However, LPS bees took much longer to learn the discrimination tasks than Elastomer and Ringer bees (two-way ANOVA: F_1, 19_ = 7.44, p = 0.013; Table S1). LPS bees required more than twice as many flower visits as Ringer and Elastomer bees, on average, to reach our learning criterion of 90% visits to rewarding flowers over three consecutive visit blocks (Fig. 1B; F_2, 27_ = 7.17, p = 0.0032). Task performance did not differ between Elastomer and Ringer bees. In the LPS group, there was no relation between number of flowers to reach the learning criterion and day tested post-injection (F_1,9_ = 1.59, r^2^ = 0.15, p = 0.24), indicating that the degree of LPS-induced reductions in bee cognitive performance and foraging proficiency persist at the same level from 1 to 7 days post-injection (d.p.i.; see Figure S1 for d.p.i. values of bees tested in each treatment group).

#### Multitasking experiment

**Figure 2:**
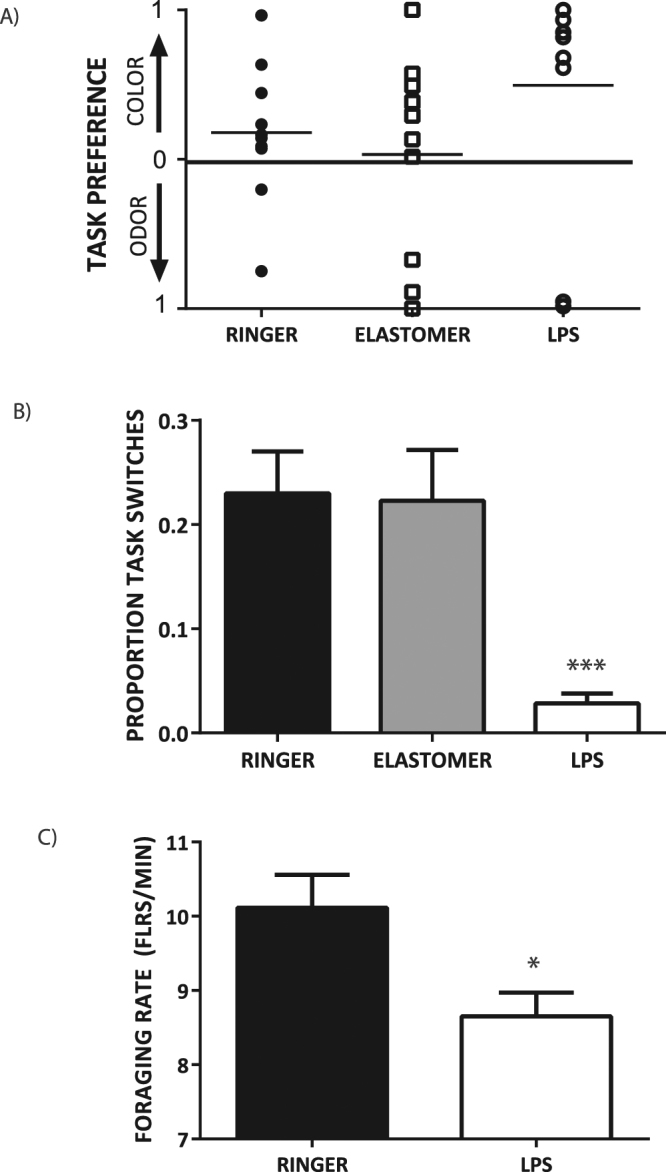
LPS-challenged bumblebee foragers have reduced cognitive flexibility and reward intake rates. Individual task preferences (**A**), mean proportion of task switches (**B**), and mean foraging rates (**C**) of Ringer, LPS, and Elastomer bees on a mixed array containing color and odor discrimination tasks. Task preference values range from 0 (no preference) to 1 (absolute preference). Foraging rates represent the number of rewarding flower types visited per minute of foraging time. Means are shown +/−SE. *P < 0.05; ***P < 0.001.

All bees from the Ringer group (10/10), 8/10 bees from the Elastomer group, and 5/10 bees from the LPS group successfully completed both color and odor discrimination tasks on the multitasking array at least once over the testing period (Fig. 2A). However, LPS bees showed a strong preference for performing the color task compared to Elastomer and Ringer bees (Mean proportion of color task choices +/−SE: LPS = 0.89 +/− 0.03; Elastomer = 0.52 +/− 0.09; Ringer = 0.59 +/− 0.06; one-way ANOVA: F_2, 22_ = 10.66, p = 0.0006). Of the remaining single-tasking bees, 1 Elastomer and 3 LPS bees specialized on the color task, and 1 Elastomer and 2 LPS bees specialized on the odor task. When single-tasking bees were subsequently forced to switch to performing the alternative task after the testing period by removing the rewarding flowers of preferred task from the multitask array, 2/2 Elastomer and 4/5 LPS bees performed the alternative task with 90% accuracy (we did not have post-test data for one LPS bee due to a technical error), indicating that bees chose task specialization as a foraging strategy despite having information on both tasks available in memory.

Despite the fact that most LPS bees successfully completed both color and odor tasks during the testing period, individuals rarely switched from one task to the other (Fig. 2B). Mean (+/−SE) task-switch frequency for LPS bees that performed color and odor tasks was 0.04 +/− 0.01 compared to 0.28 +/− 0.04 and 0.23 +/− 0.04 for bees in Elastomer and Ringer groups, respectively. There was no relation between task switch frequency and d.p.i. in the LPS group (F_1,8_ = 2.39, p = 0.16; Figs 2 and S1). Thus, as with LPS-treated bees in the single task experiment, reductions in cognitive performance persisted at the same level for up to a week post treatment.

In addition to reducing the level of task-switching behavior in bees, LPS treatment also caused a significant reduction in bee foraging rates (number of rewarding flowers visited per minute; Fig. 2C; t = 2.68 df = 18, p = 0.01), indicating that the increased cognitive task specialization of LPS challenged foragers negatively impacted the amount of nectar delivered to the colony. Since mean flower handling times (i.e. the amount of time taken to extract reward from an individual flower) did not differ between LPS and Ringer bees (mean +/− SE of pooled Ringer (4.896 +/− 0.19) and LPS (4.98 +/− 0.15) from single and multitasking experiments: Student’s t-test: t = 0.34, df = 39, p = 0.74), these results demonstrate that LPS-induced reductions in foraging proficiency were caused by a cognitive (reduced decision time) rather than motor (sickness-induced lethargy) impairment. Mean (+/−SE) foraging rates also did not differ between LPS and Ringer groups (10.73 +/− 0.46 and 10.86 +/− 0.62 flowers/min, respectively) in the single task experiment (Student’s t-test: t = 0.17 df = 20, p = 0.43), providing further evidence that LPS treatment did not impair motor function or reduce the motivation of bees to forage.

### qPCR Experiments

**Figure 3:**
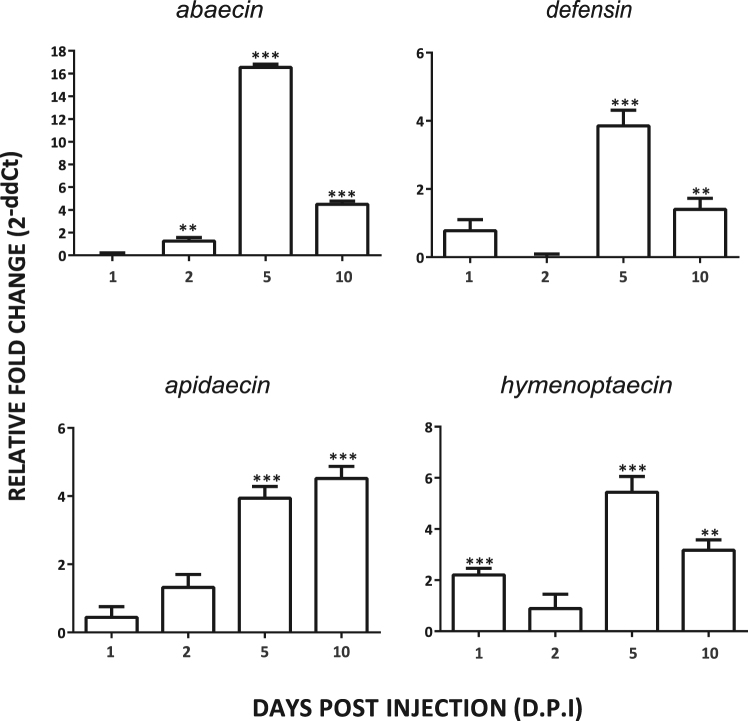
Relative expression of *apidaecin*, *abaecin*, *defensin* and *hymenoptaecin* genes as a function of days post treatment (d.p.i) for LPS-treated bees. Values represent fold increases in gene expression levels for bees in LPS treatment group relative to bees in the Ringer treatment group (denoted by the baseline value of ‘0’). Means are shown +/−SE. Fold changes were calculated using the comparative ∆∆C_T_ method. **P < 0.01; ***P < 0.001.

Figure 3 shows the fold change in AMP gene expression at each d.p.i. for LPS-treated bees relative to Ringer controls. Elastomer treatment did not increase expression levels of any of the four AMP genes above Ringer controls at any time point post-injection and therefore data were excluded from subsequent analyses (data for Elastomer bees is shown in Figure S2). All LPS bees showed up-regulation of AMP genes for up to 10 d.p.i., indicating that a single injection with LPS at a dose of 0.5 mg/mL Ringer’s solution results in the robust, long-term activation of humoral immune response pathways. However, individual AMP genes differed considerably in their duration and magnitude of increased expression, with *hymenoptaecin* (1, 5 and 10 d.p.i.) and *abaecin* (2–10 d.p.i.) genes being up-regulated for the greatest number time periods followed by *apidaecin* and *defensin* (5–10 d.p.i.). Thus, only hymenoptaecin had a range of increased expression that included all LPS bees showing cognitive impairments in our behavioral experiments (Figure S1).

## Discussion

Our study demonstrates that humoral defense responses in bumblebee foragers can produce long-term impairments to ‘higher order’ brain functions needed for the flexible and adaptive exploitation of multiple floral resources in complex sensory environments. Bees injected with LPS, a non-pathogenic elicitor of humoral immune defense response, showed substantial reductions in performance on simple color and odor discrimination learning tasks compared to Ringer control bees. This finding is consistent with previous psychoneuroimmunological studies on bees^20–22^. However, the results of our multitasking experiment significantly extend this earlier work by showing that LPS challenge also impairs cognitive processes mediating the ability of foragers to effectively manage information of multiple behavioral tasks in memory at the same time, a demand routinely placed on foragers under natural floral conditions^44^. In addition, LPS-treated foragers showed the same degree of cognitive impairment from 1 to 7 d.p.i. in both single and multitasking experiments, demonstrating that humoral immune challenge can produce rapid effects on bumblebee cognitive behavior that persist over ecologically-relevant timeframes. Importantly, we also show for the first time that cellular defense responses do not have detrimental effects on bumblebee cognition and foraging proficiency and therefore play a relatively minor role in mediating immune-cognitive system crosstalk, at least for the types of simple and complex cognitive processes examined in our study. These results collectively suggest that immune-brain connectivity in bumblebees is an inherent consequence of the humoral response to parasitic infection. Whether such connectivity is specific to activation of the Imd pathway or also includes activation of the Toll pathway (and thus would result from infection by wider range of pathogens) warrants further investigation.

Given that inter-flower decision times on the multitasking array were on the order of milliseconds to seconds, the observed increase in task repetitive behavior of LPS-challenged bees was likely caused by limitations on the capacity to hold, and/or rapidly switch between, information on color and odor tasks in working memory. Working memory can be generally defined as a memory system involving the temporary storage and processing of information required to carry out goal-driven behavioral tasks^45^. Indeed, all LPS bees specializing on a single task that were then forced to perform the alternate task did so with greater than 90% accuracy, indicating that they had learned information on both tasks during the training period and retained them in long-term memory. Limitations on working memory capacity has long been thought to underlie the tendency of bees and other pollinators to specialize on one type of flower when it makes more economic sense to visit multiple types^41,46^, a phenomenon known as ‘flower constancy’^47^. In fact, multitasking Ringer and Elastomer bees showed mean task switch frequencies that were substantially lower than those predicted from random task selection (0.23 and 0.28, respectively compared to 0.5 for random selection), suggesting that bees adopt task specialization to minimize time penalties or ‘switching costs’ associated with rapidly processing information on a different types of cognitive task. In support of this view, numerous psychological studies of voluntary task switching in humans and other vertebrates have shown that switching costs and task repetition bias (perseverative behavior) are an inherent consequence of cognitive multitasking^48,49^. It is also well-known that activation of the humoral immune defense system in vertebrate systems causes impairments to working memory, reduces cognitive flexibility, and increases repetitive behavior^34,50–52^, suggesting similar mechanisms may mediate immune-cognitive system connectivity in insects and vertebrates. Our findings highlight the potential of the bumblebee as a model system for future research on the consequences of immune-cognitive system connectivity for the ecology and evolution of plant-pollinator-pathogen interactions.

The results of our molecular experiments demonstrate that LPS challenge increases expression of all four known bumblebee AMPs for at least a 10 day period, which spans the time period over which we saw consistent cognitive deficits and reduced bee foraging proficiency in our behavioral experiments. In contrast, our Elastomer treatment, which did not affect cognitive behavior, did not increase AMP gene expression at any time point. Based on these results, we propose that humoral defense responses impair cognitive behavior due to the specific effects of AMPs on neuronal functioning in brain. In support of this hypothesis, recent genetic manipulation experiments in Drosophila have shown that overexpression of AMP genes is sufficient to impair short term memory^53^. In addition, high expression of *diptericin* in flies, which shows sequence similarity with bee-specific *hymenoptaecin*^54^, over extended periods of time (15 days) causes neurodegeneration in the brain and behavioral defects^37^. An alternative explanation of our results is that cognitive impairments resulting from humoral challenge are caused by a general increase in AMP levels in the hemolymph, or possibly by a molecular component of the Imd pathway highly correlated with AMP production such as NF-κΒ^26^. For all of these potential mechanisms of immune-brain crosstalk, a major assumption is that AMPs and other humoral immune products can move from the hemolymph into the brain to affect neuronal processes underlying cognitive function. Although defensins, abaecins, and apdiaecins have all been shown to have the capacity to cross the blood-brain barrier^55–57^, hymenoptaecin has yet to be evaluated. However, it has been classified as a cell penetrating peptide^54^, giving it the physiological and structural potential to do so^58^. Our future research efforts will systematically manipulate AMP gene expression in the fat body as well as quantify peptide levels of the four genes, as well as their degradation rates, in brain to gain further insight into their functional role in mediating immune-cognitive system crosstalk in bumblebees.

There is major global conservation concern over the potential impact of invasive and emergent pathogens on wild bumblebee communities^5,9^. Of particular concern are exotic strains of naturally occurring bumblebee pathogens such as *Nosema bombi* and *Crithidia bombi* as well as a variety of evolutionarily novel viruses and other pathogens recently introduced through contact with honeybees^11,59–62^. Bumblebee foragers are also regularly exposed to numerous microbial organisms in floral nectar and pollen with unknown pathogenic effects^63,64^. Collectively, our results suggest that any pathogen eliciting a humoral defense response in bumblebees can render foragers less able to effectively harvest, and thus compete for, floral resources. However, it has yet to be studied if immune-brain crosstalk has long term effects on the colony growth and reproductive output (i.e., fitness). Our work clearly underscores the importance of quantifying fitness costs associated with immune-brain connections before wild bee decline can be attributed to the direct action of a specific pathogen or group of pathogens. In fact, our molecular data suggest bacterial, fungal, and viral infections eliciting expression of *hymenoptaecin* and/or *abaecin* at high levels may pose the greatest threat to wild bee populations. This hypothesis is supported by recent work showing that *C*. *bombi*, a gut pathogen thought to be a significant causal factor in the rapid decline of several bumblebee species in North America^6^, both impairs cognitive behavior^15^ and elicits high expression of *hymenoptaecin*^25,65^. Given these results and those of the present study, we feel that further rigorous empirical testing of how antimicrobial peptide profiles differ between stable and threatened bumblebee species in the wild would greatly accelerate conservation efforts to establish the role of pathogenic agents in population decline.

## Materials and Methods

### Bumble bees

*Bombus impatiens* colonies were obtained from Biobest Biological Systems Canada (Leamington, ON) and connected to a small flight cage (325 × 240 × 221 cm) with a gated tube constructed from wire mesh, thus enabling control over the number of bees entering the cage. The cage was illuminated by two Ultra SunTM 6500 K (ZooMed Laboratories Inc., San Luis Obispo, USA) and two Sylvania GRO-LUX fluorescent lights. Prior to experiments, foragers collected 30% sucrose solution from several feeders placed in the center of the cage. At least two colonies were used per experiment. All newly emerged workers within a colony were marked for identification with different color combinations of acrylic paint. Colonies were directly supplied with pollen *ad libitum* to facilitate nectar foraging during experiments.

### Immune treatments

Foragers were chilled in a refrigerator at 4 °C until immobile and then randomly assigned to one of the following three experimental groups for immediate treatment: cellular immune challenge, humoral immune challenge, and control. After experimental treatment, all bees were marked with a small dot of acrylic paint on the thorax and then returned to the flight cage until testing.

#### Cellular immune challenge

Bees were injected subcutaneously with a 1 mm silicone Visible Implant Elastomer Tag (*Northwest Marine Technology*, *Inc*., *Shaw Island*, *WA*, *USA*). This material, which was originally developed for tagging aquatic animals, was ideal for non-pathogenic elicitation of a cellular immune response because it is biocompatible, easily injectable as a viscous liquid which cures inside bees as a pliable solid mass, and does not cause major tissue damage or dissipation. The colored solution and the curing agent were mixed in a 10:1 ratio as suggested by the manufacturer. The mixture was then inserted between the 4^th^ and 5^th^ abdominal segment with a 31 gauge ultra-fine insulin syringe needle (VWR BD328438). Preliminary analysis of this technique confirmed that the solution cured and stayed local to the injection site.

As Elastomer injection has not been used previously to elicit a cellular immune response in bumblebees, we conducted a preliminary experiment to determine its efficacy. Bees were randomly selected from the colony and injected with either Elastomer or 2 uL of insect Ringer’s solution (Injection control), and then housed in small microcolonies with *ad lib* sucrose and pollen. Ringer’s solution was prepared following^66^. To achieve correct pH of the solution, the bottle of dH_2_0 was sterilized prior adding the other ingredients, then pH was adjusted to 7.2.

At 24 ad 48 hours post Elastomer injection, a sterile 31-guage ultra-fine needle syringe was used to draw a hemolymph sample from between the 4^th^ and 5^th^ terga of immobilized individuals. The average volume of hemolymph obtained from an individual ranged from 4–8 uL, depending on the size of the bee. Samples were immediately transferred to a sterile 1.5 mL centrifuge tube in an ice bath and diluted 1:4 in sterile Ringer’s solution. Next, the solution was mixed well to compensate for any cell settling, and 20 µl was pipetted onto a Nexcelom disposable slide and inserted into the Cellometer Auto T4 to measure the number of hemocyte levels. Cells in four fields were counted with two technical replicates for each sample, and the values averaged. If counts varied, a third reading was taken. As expected, independent t-tests revealed that mean (+/−SE) hemocyte counts for Elastomer-treated bees were greater than Ringer controls at 24 hours (t = 2.732 df = 11, p = 0.01) and 48 hours (t = 2.346 df = 11, p = 0.02) post-injection (Figure S3), indicating that Elastomer treatment produced a robust cellular immune response in bees.

#### Humoral immune challenge

Bees were injected with 2 uL of Ringer’s solution containing lipopolysaccharide from *Escherichia coli* (LPS; *Sigma Aldrich L2755*) at a concentration of 0.5 mg/mL. LPS is well-known to be a strong non-pathogenic elicitor of a humoral immune response in bumblebees^43^. Bottles containing LPS-Ringer solution were kept at 4 °C until use. To confirm sterility, aliquots were taken from the solution and plated on LB media petri dishes along with a sterile water control, which showed no growth after 72 hours.

#### Controls

Bees were injected with 2 ul of sterile Ringer’s solution (as described above), which served as both an injection and vehicle control for Elastomer and LPS treatment, respectively. Bottles containing Ringer solution were kept at 4 °C until use.

### Behavioral assays

#### Experimental flowers

Artificial flower types (herein referred to as ‘flowers’) and arrays were modelled after^15^. Flowers were constructed by removing the cap from colored 1.5 mL Eppendorf centrifuge tube (Hamburg, GER) and fixing a 3 cm (diameter) circular collar (corolla) made of Creatology^TM^ foam (Michaels, Irving TX, USA) around the entrance of the tube. This basic design was modified to create variation in color (visual modality), odor (olfactory modality), and combinations thereof (bimodal). Color cues were varied by changing the corolla and tube to blue, orange, yellow or purple. Odor cues were varied by diluting geranium, clove, or peppermint oil in pentane (1:50) and then dispensing a 5-μl drop onto the corolla surface. Flowers associated with each cue contained either 2 uL of 30% sucrose solution (rewarding) or the same volume of distilled water (distractor), which were pipetted to the bottom of the tube. Thus, individuals had to crawl inside the tube to determine if the flower contained reward. Rewarding and distractor flowers for color and odor discrimination tasks were as follows: color task: yellow (rewarding), purple, orange (distractors); odor task: blue-geranium (rewarding), blue-clove, blue-peppermint (distractors).

#### Test array

The base of the array consisted of a horizontal block 120 × 80 × 5 cm Styrofoam covered a color print of natural foliage. At total of 90 holes were drilled in the block to hold artificial flowers, which were distributed 12 rows of 8 (12 cm apart within rows and 6 cm between rows). At total of 32 rewarding and 48 distractor (29 color and 29 odor) flowers were positioned in the 90 holes (Figure S4 (lower) shows rewarding/distractor flower arrangement for the multitasking experiment). Position of rewarding and non-rewarding flowers on test arrays did not change throughout experiments, but the cue associated with rewarding flowers changed to create single (32 color or 32 odor) and multitasking (16 color and 16 odor) experimental conditions. In Figure S4 (lower), note that rewarding flowers for color and odor discrimination tasks are interspersed within rows, giving bees the choice of performing either task as they searched for rewarding flowers. In this way, we were able to manipulate the number of tasks to be performed while holding abundance, distribution, and inter-flower flight distances between rewarding and distractor flowers constant. All flower types were replaced between test bees to control for potential influence of scent marks on choice behavior.

#### Testing Procedure

Treated bees were initially pre-trained to learn the identity of the rewarding color (yellow) and odor (geranium-scented) cue by allowing them to make three consecutive foraging runs (approximately 40 visits per run) on a pure array of each type (Figure S4, upper). The mean number of days post-treatment (d.p.i.) that pre-training and testing occurred did not differ among treatment groups (F_2, 57_ = 0.3194, P = 0.7278; Figure S1), with individuals varying from 1 to 9 d.p.i. across groups. The presentation of color and odor pre-training arrays was alternated between bees to control for potential order effects on subsequent task choice behavior. Once pre-training was completed, bees were immediately presented with the test floral array containing either one (single task experiment) or both (multitasking experiment) rewarding cues intermixed with distractor cues and the first 120 flower choices were digitally recorded for later analysis of task performance, foraging patterns, and reward intake rates. In the event that a test bee performed only one task for all 120 visits under multitasking conditions, the rewarding flowers of the preferred task were removed and flower choice behavior was recorded for an additional 20 minutes or 20 rewarding flower visits, whichever occurred first. In this way, we assessed whether or not information about the alternative task was present in memory.

#### Behavioral Data analysis

For the single task discrimination experiment, a two-way ANOVA was used to test for effects of visit block (20 flower visits/block) and treatment group proportion of rewarding flower visits. For both single and multitasking experiments, a one-way ANOVA (followed by Tukey’s post-hoc test where applicable) was used to compare mean learning rates and task-switch frequencies among Ringer, Elastomer, and LPS groups. A Student’s t-test was used to compare mean foraging rate between the Ringer and LPS groups. For single and multitasking experiments, regression analysis was used to test if impairments to cognitive performance improved over time (d.p.i.).

### Quantification of AMP gene expression levels

#### RNA extraction

Workers were extracted from colonies under red light and randomly assigned to Elastomer, LPS, or Ringer treatment groups. Workers actively engaged in nest building and brood rearing behaviors were not collected for testing because they do not forage outside the colony. Each treatment was performed as described above and then bees were held in the colony for either 1, 2, 5 or 10 days. After the holding period, individual bees were chilled to immobility in a −20 °C freezer and the legs, wings, and proboscis were dissected out and discarded. These body parts were removed to help minimize the clogging of RNA filters, which would have detrimental effects on the RNA extraction process. A −20 °C freezer was used because it rapidly placed the bees in an immobile state without freezing them and held them there for the duration of the dissection, thereby enabling bees to be processed easily and in a short amount of time. The remainder of the body was then immediately stored in a −80 °C freezer. This sample preparation procedure (from removal to placement in the −80 °C freezer) was completed in less than 10 minutes in order to minimize RNA degradation.

Samples were removed from the −80 °C freezer and RNA was extracted immediately from the body by pestle homogenization with Trizol (Life Technologies, Carlsbad CA, USA) following directions for RNA isolation and phase separation from the manufacturer. Manufacturer suggestions for tissue lysing were followed despite some samples being slightly larger than the recommended 50–100 mg of tissue mass. A minor alteration of adding Trizol in smaller subsequent additions (500 uL, 250 uL, and 250 uL) allowed for pestle homogenization room without spilling the sample yet still reaching a final volume of 1 mL. Soft tissues were completely lysed and analysis post extraction showed high concentration of pure RNA. The aqueous phase containing RNA was collected after chloroform phase separation, then purified using PureLink RNA Mini Kit (Life Technologies, Carlsbad CA, USA) ending with a 30 uL elution step. Concentration and purity ratios were measured using a Nanodrop spectrophotometer (ThermoFisher Scientific, Waltham MA, USA) and samples were diluted in molecular-grade RNAse-free and DNAse-free water to a final concentration of 100 ng/uL. Purified RNA was stored at −80 °C until use.

#### Reverse Transcription to cDNA

cDNA was synthesized from RNA by reverse transcription using a High-capacity cDNA Reverse Transcription Kit (Life Technologies, Carlsbad CA, USA) following the supplier’s recommendations. A master mix of 2.0 uL buffer, 0.8 uL dNTPs, 2.0 uL primer, 1.0 uL reverse transcriptase, and 4.2 uL of water was prepared for the total number of samples. Then 10 uL (or 1 ug) of RNA was added for each sample. The thermocycler was run following 25 °C × 10 min, 37 °C × 120 min, 85 °C × 5 min, and a final 4 °C hold. cDNA was then diluted to 1:10 in sterile water (molecular-grade RNAse and DNAse-free) and stored at −20 °C.

#### Primers and qPCR analysis

Four known bumblebee antimicrobial peptides (abaecin, defensin, apidaecin, and hymenoptaecin) were selected for transcription level analysis. An internal control or ‘housekeeping’ gene (40S ribosomal protein S5 (RPS5)) was also chosen for normalization of the qPCR results. This housekeeping gene was selected because it has been shown to have consistent temporal expression regardless of infection status^67^. NCBI identifications of the *Bombus impatiens* sequences of AMP and RPS5 genes are as follows: abaecin LOC100749486, defensin LOC100744436, apidaecins type 73-like LOC100745464, hymenoptaecin-like LOC100740362, and 40 S ribosomal protein S5 LOC100741947.

Forward and reverse primers were designed to flank gene sequences and create amplicons around 100 bp in length for each of the five genes (Table 1). Lyophilized primers were reconstituted to stock 100 µM solutions in molecular grade water which was stored at −20 °C. Prior to use, working 20x primer pair solutions were prepared by farther diluting both forward and reverse primers to 10 uM for each gene (10 uL 100 uM forward, 10 uL 100 uM reverse, and 80 uL molecular-grade water) which was also stored at −20 °C. Accuracy of primers was confirmed using standard PCR.

**Table 1:** Primer information for antimicrobial peptides genes *abaecin*, *defensin*, *apidaecin*, and *hymenoptaecin* and housekeeping gene *40S ribosomal protein S5* in qPCR analysis. Forward ‘F’ and reverse ‘R’ primer sequence are shown in the 5′–3 orientation. Primer and fragment lengths are given in number of nucleotides. GC is the percentage of guanine and cytosine bonds. Tm is the melting temperature in degrees Celsius.

Gene Name	NCBI ID	Primer Sequence (5′–3′)	Primer Length (nts)	GC (%)	T_m_ (°C)	Fragment Length (nts)
*abaecin*	LOC100749486	F: ATGAAGGCAGTAATGTTTATTTTCA^73^	25	28	60	114
		R: TGGGAAGCTTGGAAACGGTTTAGAT	25	44	68	
*defensin*	LOC100744436	F: CAACTGTCTCAGCATGGGCAAAG^74^	23	52	69	81
		R: AGATCCTTGAAGTTGGTCTTGC	22	45	65	
*apidaecins type 73-like*	LOC100745464	F: ATGAAGAACTTTATCTTCGCCATTC	25	36	63	127
		R: TTGGTTCTGGTTCTGGTCCAGCTT	24	50	71	
*hymenoptaecin-like*	LOC100740362	F: TTTTCGACGACACGCCGACCCT	22	59	74	95
		R: GTTGATGATAATCGACGTCCAAGGA^74^	25	44	66	
*40 S ribosomal protein S5*	LOC100741947	F: GAGAAGATTCCACGCGTATTGG^74^	22	50	66	129
		R: TACGGAATGCGGCTTCCCTAGCA	23	57	73	

#### qPCR reactions

qPCR was performed on an 7500 Fast System SDS Real-Time PCR System from Applied Biosystems (Foster City CA, USA). A master mix of 10 uL PowerUp SYBR Green Master Mix (Life Technologies, Carlsbad CA, USA), 1 uL of the working paired primer solution (one gene at a time), and 4 uL of water was created for all samples. cDNA was diluted again 1:5 (2% of original reverse transcription product) and was pipetted to the bottom of a 96-well plate. 15 uL of the master mix was added to each well. Each plate was designed to minimize interplate variation in threshold cycle (CT) values by running two technical replicates of every sample within all treatment groups (n = 5 bees in each of Ringer, Elastomer, and LPS groups) for a single gene-time point combination plus a contamination control. This ‘sample and treatment maximization’ plate design^68^, which has been used in previous studies of AMP expression levels in bumblebees^25^, was used to enable effective statistical comparisons of Elastomer and LPS treatment groups relative to Ringer control at each time point. The thermocycler was run using the following sequence along with a dissociation curve to check for off-target amplification: 95 °C × 20 sec, [95 °C × 3 sec, 60 °C × 30 sec] followed by 40 cycles of 95 °C × 15 sec, 60 °C × 60 sec, 95 °C × 15 sec, and 60 °C × 15 sec. Fluorescence curves were inspected visually for appropriate dissociation in order to ensure against primer artefacts and to check for DNA contamination. C_T_ thresholds were set automatically with occasional minor adjustments to areas of more gradual slope and clear separation. These data were then saved for subsequent analysis.

#### Analysis of AMP Expression Levels

We used the comparative ∆∆C_T_ method^69^ to determine if treatment with Elastomer and LPS increased transcription levels of *abaecin*, *defensin*, *apidaecin*, and *hymenoptaecin* antimicrobial peptide genes relative to Ringer controls at 1, 2, 5, and 10 d.p.i.. A total of five bees were individually analyzed per treatment group per time point (60 bees total), with two technical replicates taken per gene for each bee. This number of biological and technical replicates is consistent with other studies examining expression levels of immune gene expression in bees^70–72^. We first normalized the threshold cycles (Ct) value of each AMP gene against the housekeeping gene RPS5 (Ct_AMP_ − Ct_RPS5_), resulting in a set of individual ∆C_T_ values for bees in Elastomer, LPS and Ringer groups at each time point. The two ∆CT technical replicates for each individual were then averaged to produce a single ∆CT value per individual. Next, we normalized mean ∆C_T_ values of individuals in the Elastomer and LPS groups against the mean ∆C_T_ value of individuals in the Ringer group at each time point (∆C_TTreatment_ − ∆C_TRinger_), yielding a ∆∆C_T_ value for each group-time point combination. Finally, we determined the relative fold change in AMP gene expression for each group-time point combination using 2^−(∆∆CT)^ − 1, which set baseline value (i.e. no difference in AMP expression compared to the Ringer group) to ‘0’. We used a one sample t-test with a Bonferroni correction for multiple comparisons to test whether or not relative fold changes for each AMP-time point combination were above baseline value.

### Data availability

All data generated or analyzed during this study are included in this published article (and its Supplementary Information files).

## Electronic supplementary material

Supplementary Information
