# Supplementary material for: Immune-cognitive system connectivity reduces bumblebee foraging success in complex multisensory floral environments
Source: Sci Rep. 2018 Apr 13;8:5953. doi: 10.1038/s41598-018-24372-5 (PMC5899130; doi:10.1038/s41598-018-24372-5)

Supplementary Information

Immune-cognitive system connectivity reduces bumblebee foraging success in complex multisensory floral environments.

Melissa W. Mobley[1] and Robert J. Gegear[1,*]

* correspondence to rgegear@wpi.edu

Table S1. Two-way ANOVA results for performance of bees on a simple discrimination learning task subsequent to Ringer, Elastomer and LPS treatment. Each block represents 20 flower choices.

ANOVA table	SS	DF	MS	F (DFn, DFd)	P value
Interaction	0.03097	5	0.006195	F (5, 95) = 1.291	P = 0.2741
BLOCK	0.2877	5	0.05753	F (5, 95) = 11.99	P < 0.0001
TREATMENT	0.1032	1	0.1032	F (1, 19) = 7.440	P = 0.0134
Subjects (matching)	0.2636	19	0.01387	F (19, 95) = 2.892	P = 0.0004
Residual	0.4557	95	0.004797		

Figure S1. Box and whisker plot of day tested post injection (d.p.i) for bees in Ringer, Elastomer, and LPS groups. Filled circles represent values for individual bees.

Figure S2. Relative expression of *apidaecin*, *abaecin*, *defensin* and *hymenoptaecin* genes as a function of days post treatment (d.p.i) for Elastomer-treated bees. Values represent fold changes in gene expression levels of bees in the Elastomer treatment group relative to bees in the Ringer group (denoted by the baseline value of ‘0’). Fold changes for each group were calculated with the $\Delta\Delta C_T$ method.

Figure S3. Mean (+/-SE) hemocyte counts for Ringer and Elastomer bees at 24 and 48 hours post treatment. Numbers = sample size for each group. *, p<0.05

Figure S4. Training and testing arrays used to assess cognitive performance of bees. Individuals were initially allowed to make consecutively visits an array of yellow flowers followed by an array of blue geranium-scented flowers, and then immediately presented with a mixed array containing both one (single task experiment) or both (shown; multitasking experiment) rewarding types intermixed with non-rewarding distractor flowers. Numbers denote flower odor; 1 = clove; 2 = peppermint; 3 = geranium. Box denotes position of rewarding flowers. Position of rewarding and distractor flowers did not change across experiments and bees. For single tasks experiments, training was the same but all rewarding positions had either yellow or blue geranium-scented flowers. Background image by M.W. Mobley.

Figure S1

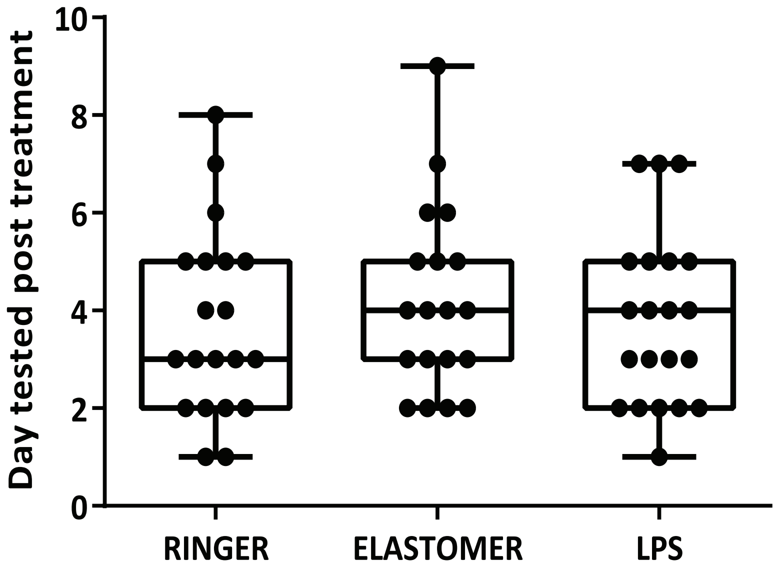

Figure S2

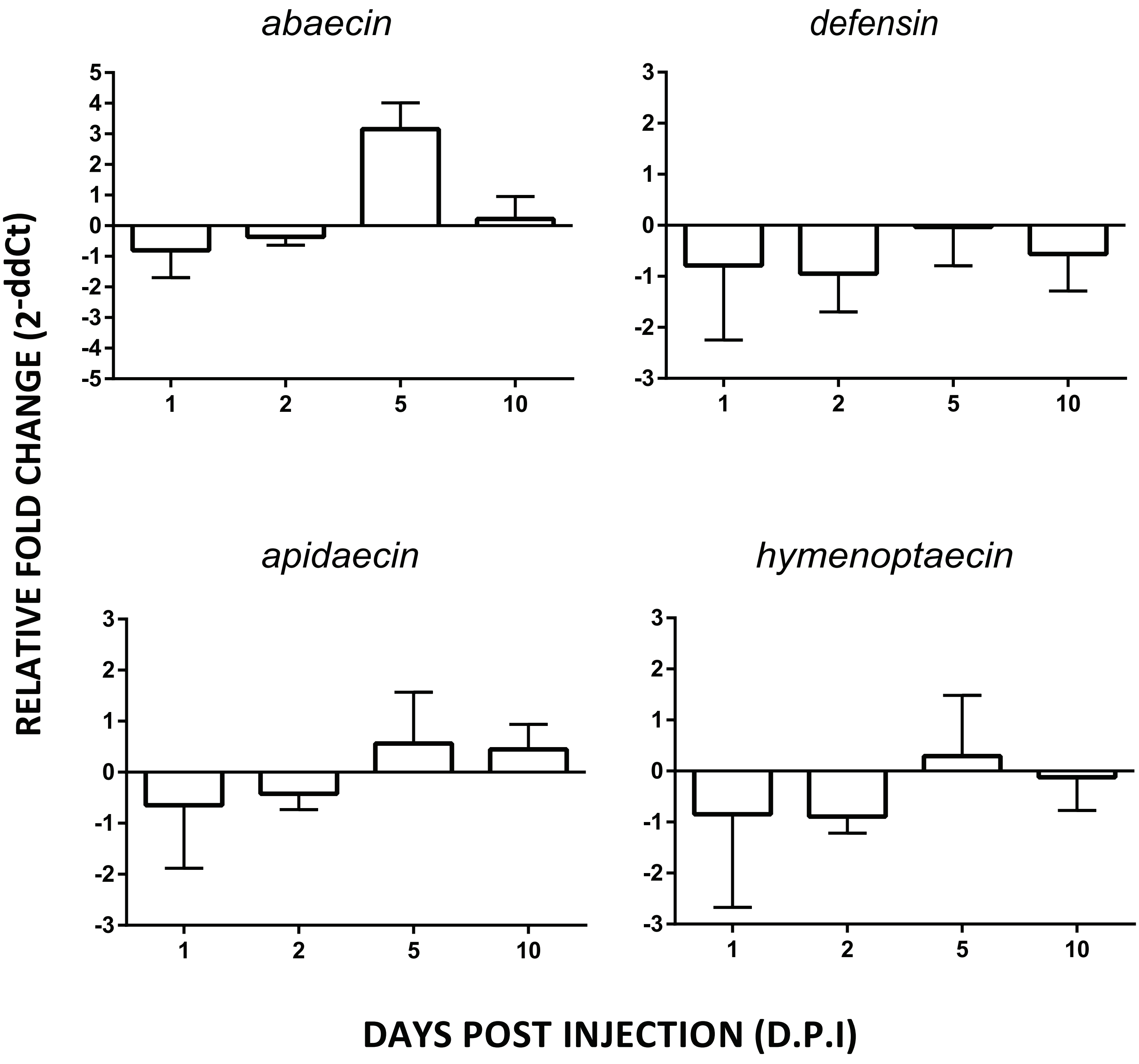
abaecin
defensin
apidaecin
hymenoptaecin
RELATIVE FOLD CHANGE (2-ddCt)
DAYS POST INJECTION (D.P.I)
5
4
3
2
1
0
-1
-2
-3
-4
-5
1
2
5
10

Figure S3

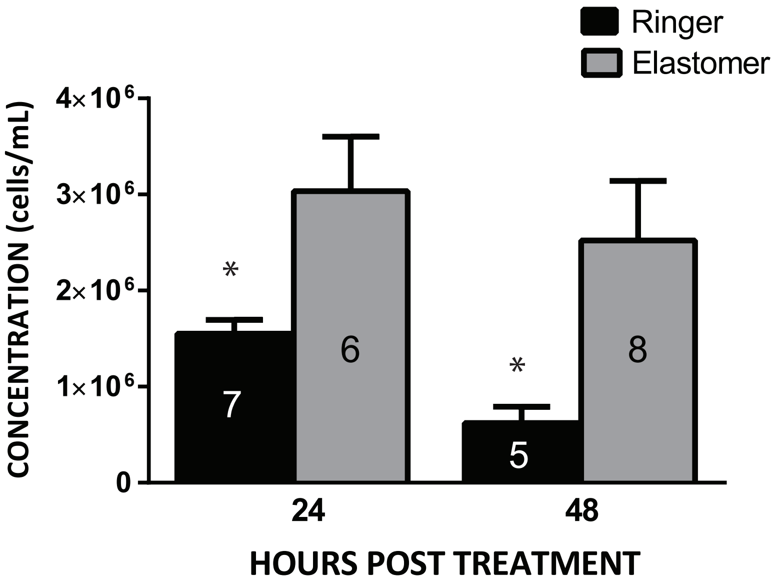
Ringer
Elastomer
4×10^6
3×10^6
2×10^6
1×10^6
0
CONCENTRATION (cells/mL)
*
*
7
6
5
8
24
48
HOURS POST TREATMENT

Figure S4

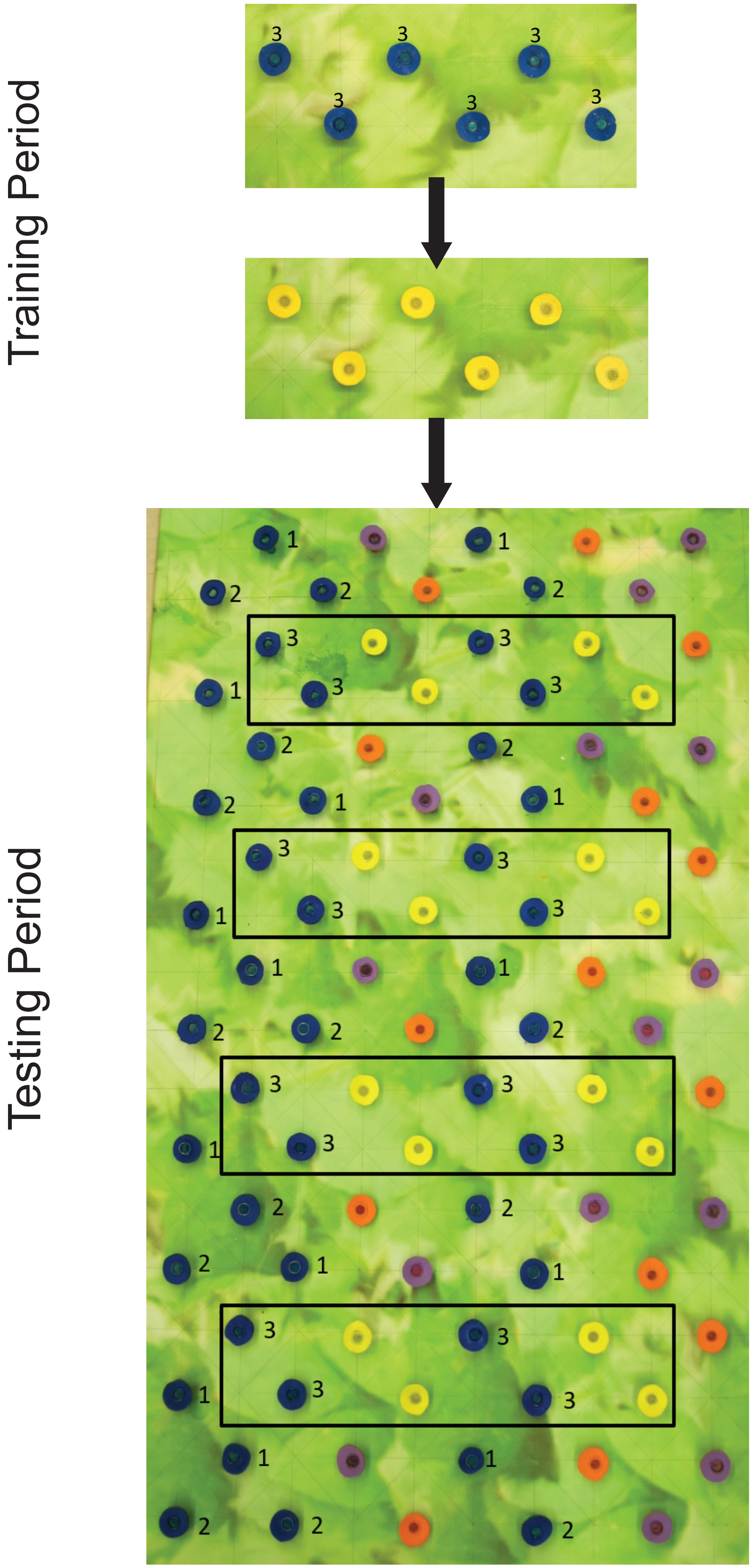

Supplement: Supplementary file 1 — Supplementary Information [file 41598_2018_24372_MOESM1_ESM.pdf]
